# Polypyrrole and Graphene Nanoplatelets Inks as Electrodes for Flexible Solid-State Supercapacitor

**DOI:** 10.3390/nano11102589

**Published:** 2021-09-30

**Authors:** Antonella Arena, Caterina Branca, Carmine Ciofi, Giovanna D’Angelo, Valentino Romano, Graziella Scandurra

**Affiliations:** 1 Department of Engineering, University of Messina, 98166 Messina, Italy; cciofi@unime.it (C.C.); gscandurra@unime.it (G.S.); 2 Department of Mathematical and Computational Sciences, Physical Science and Earth Science, University of Messina, 98166 Messina, Italy; caterina.branca@unime.it (C.B.); gdangelo@unime.it (G.D.); valentino.romano@unime.it (V.R.)

**Keywords:** graphene nanoplatelets, polypyrrole, conducting inks, flexible supercapacitor

## Abstract

Flexible energy storage devices and supercapacitors in particular have become very attractive due to the growing demand for wearable consumer devices. To obtain supercapacitors with improved performance, it is useful to resort to hybrid electrodes, usually nanocomposites, that combine the excellent charge transport properties and high surface area of nanostructured carbon with the electrochemical activity of suitable metal oxides or conjugated polymers. In this work, electrochemically active conducting inks are developed starting from commercially available polypyrrole and graphene nanoplatelets blended with dodecylbenzenesulfonic acid. Films prepared by applying the developed inks are characterized by means of Raman measurements, Fourier Transform Infrared (FTIR) analysis, and Atomic Force Microscopy (AFM) investigations. Planar supercapacitor prototypes with an active area below ten mm^2^ are then prepared by applying the inks onto transparency sheets, separated by an ion-permeable nafion layer impregnated with lithium hexafluorophospate, and characterized by means of electrical measurements. According to the experimental results, the devices show both pseudocapacitive and electric double layer behavior, resulting in areal capacitance that, when obtained from about 100 mF⋅cm^−2^ in the sample with polypyrrole-based electrodes, increases by a factor of about 3 when using electrodes deposited from inks containing polypyrrole and graphene nanoplateles.

## 1. Introduction

Over the past few decades, there has been an increasing research interest in the field of energy storage systems and, among them, a great deal of attention has been focused on supercapacitors [1,2,3,4]: devices that combine a high power density and a fast charging rate with a simple design that enables them to be flexible, stretchable, and bendable [5,6,7], qualities that make them particularly suited for wearable electronic applications [8,9,10]. Supercapacitors, which consist of a couple of electrodes impregnated with an electrolyte and spaced by an ion-permeable separator, have performances, electric behavior, and even working principles that change depending on the nature of the electrodes, of the medium interposed between the electrodes, and of the electrolyte. In particular, the choice of the electrode is a key element, as the main energy storage mechanism of any supercapacitor depends on the structural, morphological, charge-transport, and chemical properties of the electrode material it uses. When a couple of conducting electrodes impregnated by an electrolyte are bridged by a porous medium that allows ion diffusion, depending on the valence electron density of the states of the electrodes, mobile cations or anions accumulate at each of the electrode–electrolyte interfaces. The higher the electrode surface area is, the more extended the available surface for the ions to accumulate on is. As the charge accumulated at the electrodes changes in response to an externally applied voltage, the system works as an electrical double-layer capacitor (EDLC). Such a capacitive behavior can be easily evidenced by electrical measurements, as the current though an EDLC, recorded in response to a train of triangular voltage pulses and reported as a function of the applied voltage, ideally forms rectangularly shaped cycles, while the voltage charge–discharge curves recorded at a constant current are expected to be a linear function of time. This is usually found to be the case in supercapacitors with a high surface area and with highly conducting electrodes consisting of nanostructured carbon materials, including graphene and carbon nanotubes [11,12,13]. However, if the employed electrode is electrochemically active, as is the case with electrodes based on ruthenium oxides, iron oxides and hydroxides, and binary metal oxides [14,15,16,17] or when conjugated polymers such as polypyrrole, polyaniline and poly(3,4-ethylenedioxythiophene) are involved [18,19,20], fast and reversible redox reactions occur at the electrode surface, which may (in the case of pseudo-faradaic behavior) or may not (in the case of intercalation reactions) involve structural phase transitions experimented upon by the electrode itself. Both the redox and intercalation processes occurring at the electrodes are driven by the externally applied voltage and may show themselves as current peaks in the current–voltage loops recorded in response to trains of triangular voltage pulses. While the distinction between ideal EDLCs and ideal pseudo-capacitors is straightforward, reality is far more complex in the sense that the two different kinds of mechanisms often coexist: even in the case of supercapacitors based on graphene when they are classified as EDLCs, the capacitive effects that can also arise from redox processes involving electrochemically active functional groups at the graphene surface cannot be excluded. From an oversimplified point of view, it is commonly acknowledged that supercapacitors using nanostructured carbon powder have superior stability and lower capacitance compared to faradaic pseudo-capacitors. These latter ones, in addition to the intrinsic degradation of the electrode’s performance with time due to the irreversible characteristic of the redox processes, may suffer due to the fact that the electrode material may have a lower conductivity when it is based on metal oxides, hydroxides, and binary metal oxides, while in the case of electrodes based on electrochemically active conjugated polymers, the poor porosity of the polymer surface may affect the ion intercalation efficiency. An obvious strategy to achieve supercapacitors with improved performances is therefore the use of hybrid electrodes, commonly nanocomposites, that combine the excellent charge transport properties and high surface area of nanostructured carbon with the electrochemical activity of suitable metal oxides or conjugated polymers. Following one of the most popular choices in the scientific literature, the use of graphene and polypyrrole, in this paper, we show that electrochemically active films based on either polypyrrole, or graphene, or a mixture of the two can be applied using refillable pens filled with water inks consisting of commercially available unsoluble polypyrrole powder, graphene platelets, and dodecylbenzenesulfonic acid. When interfaced to nafion separating membranes impregnated with lithium hexafluorophospate in a planar capacitor configuration, the electroactive film surface undergoes faradaic processes, as evidenced by the presence of well-defined current peaks in the current–voltage cycles, yielding specific capacitance in the range of one hundred mF/cm^2^. 

## 2. Materials and Methods

**Figure 1:**
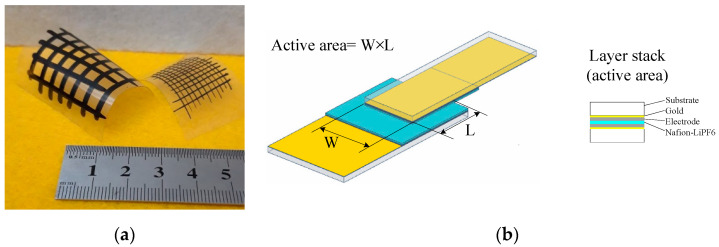
(**a**) Conducting patterns on transparency sheets that were handwritten using pens filled with the PPy:DBSA ink (the sample with thicker lines, viscosity 2.58 mPa⋅s) and the PPy-GNP:DBSA ink (the sample with thinner lines, viscosity 4.11 mPa⋅s); (**b**) schematic view of the supercapacitor structure. The substrate (Substrate) was cut out from a copier grade transparency sheet on top of which a gold layer (Gold) was thermally evaporated. Electrodes (Electrode) were applied using rechargeable pens filled with inks prepared as described in the above section. A nafion membrane impregnated with lithium hexafluorophospate was used as a separator (Nafion-LiPF6).

Ionic surfactant dodecylbenzenesulfonic acid (DBSA) 70% in isopropanol, graphene nanoplatelets (GNP), doped polypyrrole (PPy) loaded with 20% carbon black, and lithium hexafluorophospate (LiPs) were purchased from Aldrich (Milan, Italy), while nafion (5% alcohol dispersion) was provided by Ion Power. All of the materials were used as received. Based on our previous experience in the case of graphene-DBSA only [21], we knew that the electrical conductivity, morphology, and electrochemical activity of the materials obtained from mixtures of conducting nanostructured carbon and surfactants depend on the ratio between the components of the blends. Here, we chose to blend either PPy powder, GNP, or both in DBSA at a ratio of 1:5 by weight as a compromise to achieve good dispersion and good filming properties at the same time [21]. The PPy powder was added to the DBSA in isopropanol at a ratio of 1:5 by weight, and the mixture was vigorously blended until a homogenous viscous PPy:DBSA past was obtained. A three-component blend, PPY-GNP:DBSA, was then obtained by dispersing a mixture of GNP and PPy (at a ratio 1:5 by weight) into the DBSA (one part of the mixed GNP-PPy powder into five parts of DBSA by weight). Deionized water was added to the mixtures (10 cc of water per 300 mg of starting mixture), and after few hours of ultrasonication, water-based inks suitable for refillable pens were obtained. The developed inks, which were stored in glass flasks, were found to be stable for a few days, after which they could be completely revived by a few minutes of stirring by means of a magnetic agitator. The filtered inks were applied both on transparency sheets and on silicon substrates; they were slowly dried and then held at 70 °C for a few minutes to remove the residual solvent, obtaining conducting films suitable for use as electrodes. The surface quality and conductivity of the films were found to be quite unaffected by the inks’ ageing. However, at the present stage, we could not provide a quantitative characterization of their mechanical properties: the PPy:DBSA and the PPy-GNP:DBSA films seemed to have quite good adherence to the transparency sheets chosen as substrates, and there was no evidence of fractures or detachment with bending, as observed in the samples shown in Figure 1a.

Ion-doped nafion membranes were drop-deposited from the mixtures prepared, starting with 10 mL of ethanol, to which 300 mg of LiPs and 3 mL of alcoholic nafion dispersion were added while being stirred.

Solid-state planar capacitors were developed using transparency sheets as substrates, onto which a gold film had previously been deposited by thermal evaporation in vacuum conditions. The electrodes were applied at the extremities of a pair of gold coated transparency strips through pens (either AeroColor Professional Liner N.2 by Schmincke or 221EM 4 mm round tip by Molotow) filled with the inks prepared as described in the preceding section. After drying in air and after a few minutes of storage at about 60 °C to allow the evaporation of the residual solvent, the nafion–LiPs blend was drop-deposited onto the electrode on one of the strips. The drop-deposited blend, which had been flattened with a spatula, was left to dry slowly, yielding an ion permeable membrane impregnated with lithium salt on the top of the electrode. At this stage, the second strip was superimposed to the underlying strip (with the electrode facing the electrode on the first strip) and was stored at about 60 °C for a few tens of seconds in order to achieve adherence between the two pieces. A schematic view of the developed device is shown in Figure 1.

## 3. Characterization

The morphology of the developed films was investigated by AFM measurements, which were conducted by means of a Nanosurf FlexAFM equipped with a C3000 controller (Nanosurf AG, Liestal, Switzerland). Powder samples for Raman and FTIR measurements were prepared as dispersions into a 50:50 (in volume) solvent mixture made up of distilled water and ethanol. The dispersions were then sonicated for 30 min and were then drop-casted onto a Si grid and Si window (for Raman and FTIR measurements, respectively) on a hot-plate at 100 °C. The PPy:DBSA and PPy-GNP:DBSA samples were prepared by casting the functional inks on the Si grid and Si window (for Raman and FTIR measurements, respectively).

Raman spectra were recorded at room temperature using a LabRam HR800 (Horiba Italia SRL, Rome, Italy) spectrometer with a 532 nm excitation wavelength, an 1800 gr/mm grating, and a liquid nitrogen-cooled CCD camera. The measurements were performed with a 50× objective and a laser power of 1 mW to avoid any heating-induced degradation effects.

The FTIR spectra were recorded at room temperature in transmission mode with a Bruker Vertex 80V FTIR spectrometer (Bruker Italia Srl, Milan, Italy). For each spectrum, over 128 scans with a 2 cm^−1^ resolution were performed on average. Each spectrum was recorded by subtracting the background signal, which was recorded at the beginning of each measurement.

The electrical measurements were performed at about 27 °C and at a relative humidity of 58% by means of a Keithley 2400 source meter (Tektronix, Beaverton, OR, USA).

## 4. Results and Discussion

**Figure 2:**
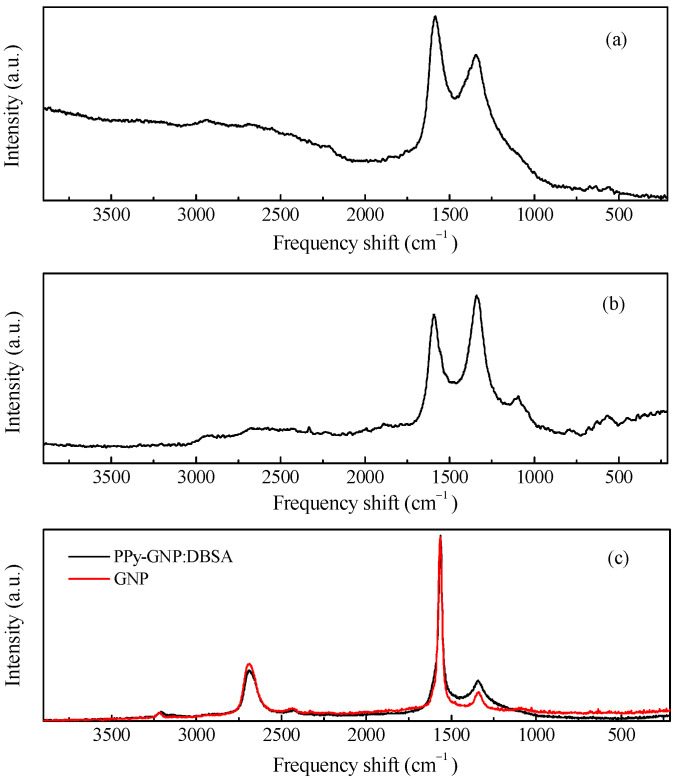
Raman spectra of the PPy powder (**a**), PPY-DBSA (**b**), and PPy-GNP:DBSA compared to GNP powder (**c**).

**Figure 3:**
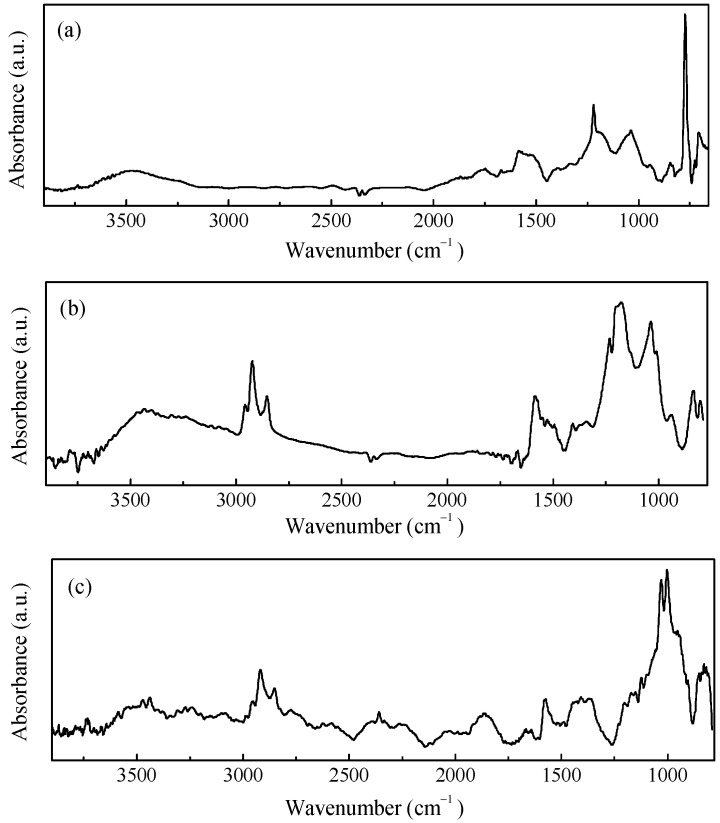
IR spectra of the PPy powder (**a**), PPY-DBSA (**b**), and PPy-GNP:DBSA (**c**).

Due to its ability to undergo electrically driven reversible transitions between its oxidized and reduced forms, polypyrrole is by far the most popular conjugated polymer that can be used as a component in electrodes for electrochemical energy storage devices [22,23,24]. The polypyrrole employed here (from now on referred to as PPy), is an insoluble powder of electrically conducting polypyrrole doped by a proprietary organic sulfonic acid for and that has been loaded with 20% carbon black to be used either as a compressed pellet or to be somehow dispersed into a host matrix and then processed as a film. The PPy powder was characterized by means of Raman and FTIR measurements, the results of which are shown in Figure 2a and Figure 3a. The two main contributions found in the Raman spectrum of Figure 2a, which are positioned at about 1339 cm^−1^ and 1592 cm^−1^, are due to the PPy ring and to the C=C backbone vibrations, respectively [25]. Moreover, other components are observed at approximately 570 cm^−1^ and 1104 cm^−1^, the former of which is related to N-H modes, and the latter of which is ascribable to C-H modes [26]. The IR spectrum shown in Figure 3a, reveals the most common infrared contributions of polypyrrole, including the band at about 1540 cm^−1^ that comprises the C=C stretching mode of the pyrrole ring (at approximately 1560 cm^−1^ and 1460 cm^−1^, respectively) and the bands at about 1185 cm^−1^ and 1037 cm^−1^, which are related to C-H bending [27]. The weak contributions observed in the region from 3000 cm^−1^ to 3750 cm^−1^ is usually associated with the O-H, C-H, and N-H modes because of the adsorption of water molecules and hydrocarbons. Figure 2b and Figure 3b show the Raman and infrared spectrum of films deposited using a rechargeable pen filled with an ink containing PPy and DBSA at a ratio 1:5 by weight. As it can be noticed, the FTIR spectrum of the PPy:DBSA film reported in Figure 3b, shows most of the features observed in the PPy sample spectrum (Figure 3a) but with rather different shapes and with a different intensity ratio and some additional peaks such as those at 2958 cm^−1^, 2924 cm^−1^, and 2853 cm^−1^, which are ascribable to DBSA [28]. By comparing the Raman spectra of Figure 2a,b it emerges that after the insertion of DBSA, the spectral features due the N-H and C-H contributions that are clearly visible in the PPy spectrum weaken, and the two main peaks of the spectrum in Figure 2b (observed at approximately 1341 cm^−1^ and 1584 cm^−1^, respectively), are slightly shifted compared to those of Figure 2a. Such spectral changes, together with the observation that no band ascribable to DBSA is identified in the PPy:DBSA Raman spectrum, suggest that DBSA, which does not act as a dopant, being the already doped PPy powder used in the current study, may somehow interact with PPy [29], for instance, by forming noncovalent bonds with the polymer backbone, as reported elsewhere [30,31]. The Raman spectrum of a film deposited from the three-component ink, PPy-GNP:DBSA, shown in Figure 2c, closely resembles that of pristine GNP powder, the D band in particular, which can be observed at approximately 1340 cm^−1^ and is related to the breathing mode of the carbon atoms with sp2 hybridization, which provides information about the presence of defects in the honey comb structure because a defect is required for that specific vibrational mode [32]. The G peak (found at about 1370 cm^−1^) stems from a phonon mode with E2g symmetry at the Brillouin zone center [33,34] and has a frequency close to that of the D’ mode (~1610 cm^−1^), a double resonance process arising from its activation in defected graphene [35]. 

Finally, the 2D peak region (~2690 cm^−1^, the second order of the D contribution) is also present in the absence of the defect-activated mode [32]. While the Raman spectrum of the film deposited from the three-component ink of fig. 2c nearly coincides with that of GNP, the FTIR analysis performed on the same film, reported in Figure 3c, resulted in a complex spectrum, where the vibrational signatures of the molecular species observed in the previous spectra are to present to a greater or lesser extent and share a close resemblance to the FTIR spectrum of the PPy:DBSA film in Figure 3b.

**Figure 4:**
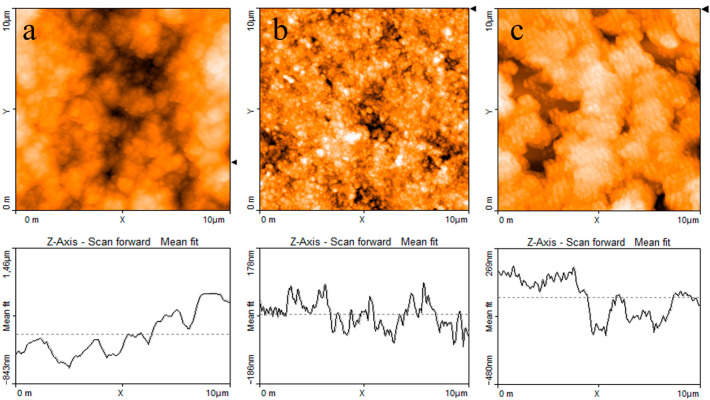
AFM images and line profiles (**a**) of the PPy powder; (**b**) a PPy:DBSA film deposited from an ink containing PPy and DBSA; (**c**) and PPy-GNP:DBSA film deposited from the ink containing both PPy and GNP. The triangular markers at the right side of each micrograph identify the position of the horizontal line along which the thickness profiles (reported at the bottom of each micrograph) are measured.

**Figure 5:**
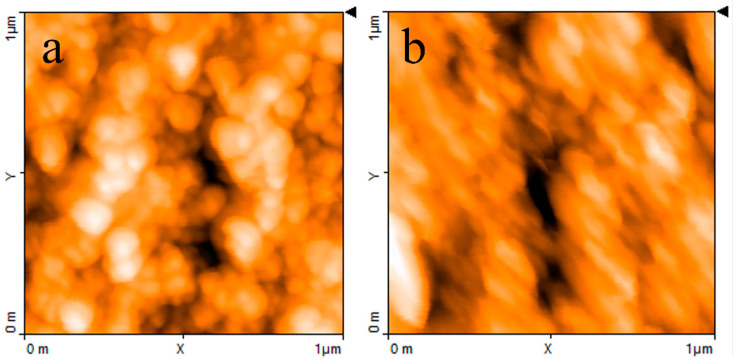
(**a**) Detailed AFM view of the sample imaged in Figure 4b and (**b**) of the sample imaged in Figure 4c.

The AFM image reported in Figure 4a shows that when the PPy powder simply pressed onto the silicon substrate, it has a granular morphology and a highly irregular profile. Figure 4b,c report the typical results of AFM measurements performed on a PPy:DBSA film and on a PPy-GNP:DBSA film. Compared to Figure 4a, the morphology of a typical film deposited from an ink containing PPy and DBSA at a ratio 1:5, as shown in Figure 4b, has a finer texture and smoother profile. This is confirmed by the detailed view of the same PPy:DBSA sample from Figure 4a, imaged over a 1 μm wide scale in Figure 5a, which reveals the presence of granules that are hundreds of nanometers wide. Although the morphology may slightly change from sample to sample, depending on the drying conditions and on the thickness of the examined films, the common feature that emerged in all of the examined PPy:DBSA samples is that when blended to the polymer at a ratio 5:1 by weight, DBSA is quite a good dispersing agent for PPy. At the same time, as the Raman spectrum suggests that DBSA seems to interact with the polymer chain, it is likely that the surfactant binds to the surface of each polymer granule, providing the link between each individual granule. In fact, while the pressed pristine PPy powder does not adhere to the substrate at all and easily disaggregates, the PPy:DBSA films deposited from the 1:5 ink onto the plastic substrates, either by means of a spatula or by direct writing trough an ink filled pen, appear to be smooth and quite compact, with good adherence to the substrate (see Figure 1a), properties that are promising in view of their use as wearable electronic. 

According to Figure 1a, PPy-GNP:DBSA films, when deposited on transparency sheets from the ink developed as previously described, also appear to be smooth, compact, and with good adherence to the substrates. On a smaller scale, the morphology of a typical PPy-GNP:DBSA film imaged in Figure 4c shows that the presence of graphene in the three-component mixture has a peculiar effect on the sample, which acquires a certain degree of self-organization. We have previously observed a similar peculiarity when examining how films deposited from mixtures containing GNP and DBSA at ratios below 1:2 and above 1:10 [21] show a tendency to organize themselves in a fairly ordered way over regions that are over a hundred micrometers in size. The PPy-GNP:DBSA film shown in the micrography of Figure 4c seems to consist of micron-sized aggregations of fairly oriented elements. These latter ones, according to the detailed view of the same sample reported in Figure 5b, have a peculiar shape that is composed of fibers that are tens nanometers wide and hundreds of nanometers long.

**Figure 6:**
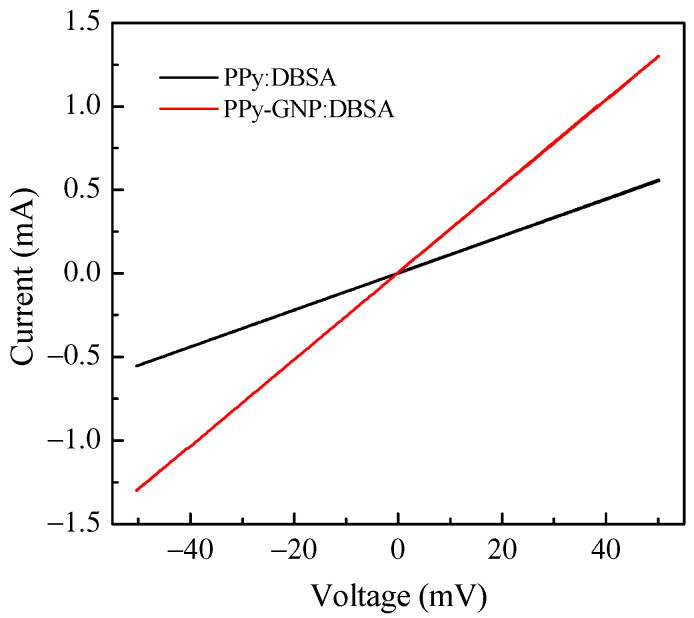
Current–voltage plots of PPY:DBSA and PPy-GNP:DBSA films.

**Figure 7:**
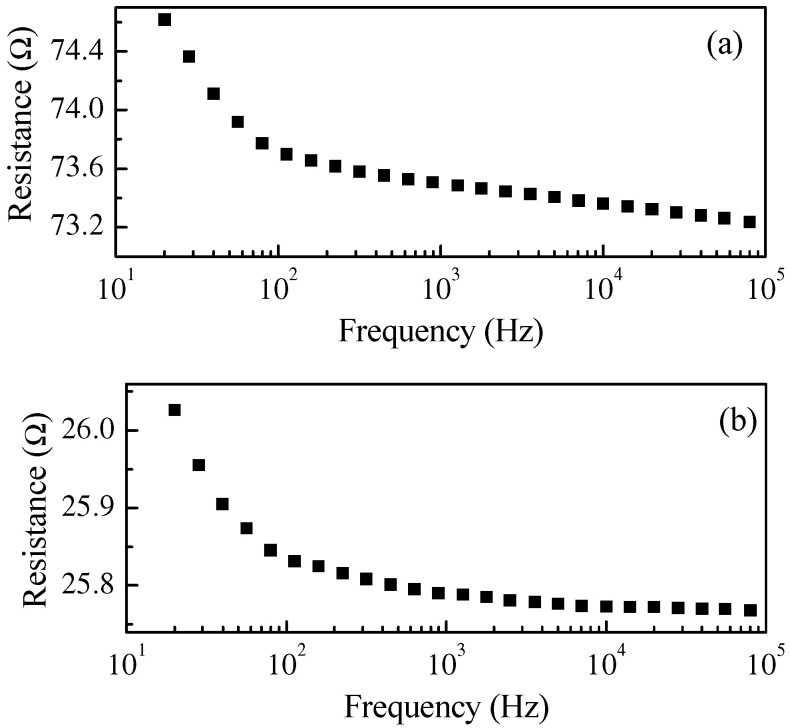
Frequency dependence of the real part of the impedance of a PPY:DBSA film (**a**) and of a PPy-GNP:DBSA film (**b**). The imaginary part of the impedance is negligible in both cases at all of the explored frequencies.

The electrical characterization of the PPy:DBSA and PPy-GNP:DBSA electrodes was performed on the films deposited from the respective inks onto a half millimeter wide gap separating parallel gold electrodes applied on transparency sheets. Figure 6 compares the results of the current–voltage measurements, which indicate that both materials have a linear ohmic behavior, with a higher slope corresponding to the third factor of lower resistance for the sample prepared from the PPy-GNP: DBSA ink. The ohmic behavior of the electrode materials is confirmed by the results of the impedance measurements performed between 20 Hz and 100 kHz, which indicated that for both the PPy:DBSA and the PPy-GNP:DBSA films, the magnitude of the imaginary part of the impedance was three orders of magnitude below the real part. The resistances vs. frequency of the same PPy:DBSA and PPy-GNP:DBSA films that Fig. 6 refers to are reported in Figure 7, and it can be noted that they decrease slightly as the frequency increases. According to the results shown in Figure 6 and Figure 7, it can be concluded that the films deposited from the three-component ink have higher electrical conductivity compared to those obtained from PPy-DBSA because of the presence of GNP. 

**Figure 8:**
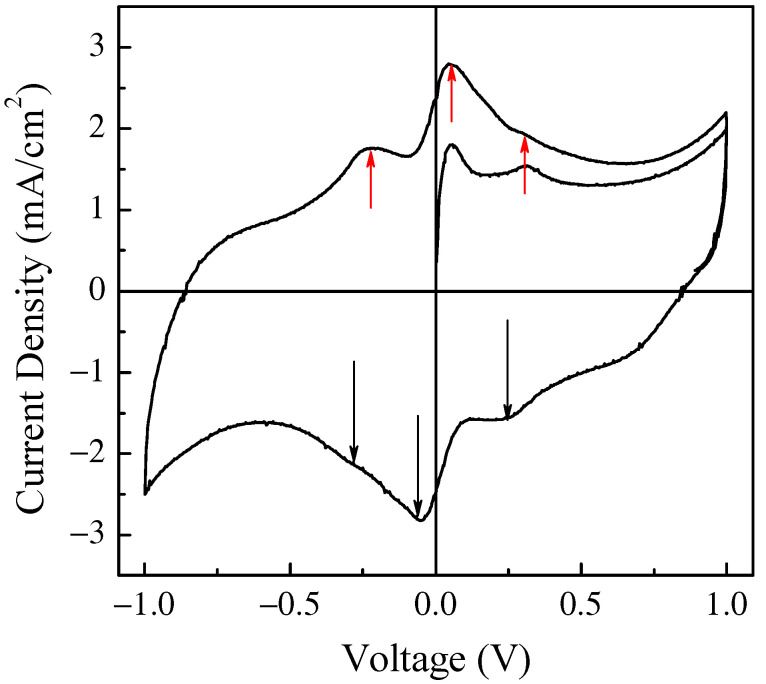
Transient phase and current density–voltage loop of a typical Au/PPy:DBSA/Nafion:LiPs/PPy:DBSA/Au cell in response to 1V amplitude triangular voltage pulses with a 300 s period and corresponding to a scan speed of ~13.3 mV/s.

The results of current density–voltage measurements performed on a typical Au/PPy:DBSA/Nafion:LiPs/PPy:DBSA/Au electrochemical device with active area of about 6 mm^2^, the schematic view of which is shown in Figure 1, are shown in Figure 8.

The current density through the device was measured in response to zero average triangular voltage pulses with a 1 V amplitude and period of 300 s, which corresponds to a voltage time rate of change of about ± 13.3 mV/s. After a transient phase, the current density starts to cycle, resembling a fairly symmetrical clockwise loop that reflects the symmetry of the device and that is far from the rectangular shape expected in the case of EDLCs. Two forward current density peaks and a shoulder, which were recorded while the voltage linearly increased with time and that can be identified by the red arrows in the figure, are observed at about −217 mV, 51 mV, and 297 mV. Two reverse current density peaks and a shoulder, which were recorded while the voltage linearly decreased a function of time, are identified by the black arrows and can be found at approximately 203 mV, −54 mV, and 281 mV. It is well known that the current–voltage loop of a capacitive device changes with the absolute value of the voltage’s time rate of change, namely with the scan speed, in a way that is related to the kind of mechanisms from which the capacitance of the device originates. In fact, the current intensity is expected to be a linear function of the scan speed in EDLCs, while the relationship between the intensity of the current peaks and the scan speed, though not necessarily linear, can provide useful information concerning the redox processes at the electrodes. Figure 8 shows the results of the current density–voltage measurements performed at different scan speeds on the same Au/PPy:DBSA/Nafion:LiPs/PPy:DBSA/Au that Figure 8 refers to. 

**Figure 9:**
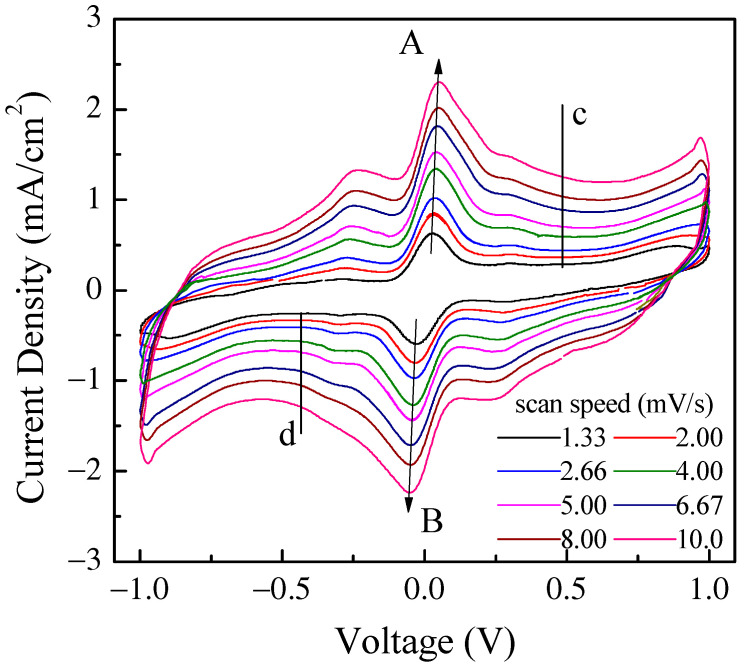
Current density–voltage loop of the same Au/PPy:DBSA/Nafion:LiPs/PPy:DBSA/Au cell that Figure 8 refers to measured at different scan speeds. The arrows mark the forward (A) and reverse (B) current density peaks, the intensity of which is reported in Figure 10a. The vertical lines marked c and d in the flat region of the inner loop identify the voltage V_c_ (V_d_) at which the forward (reverse) current density data displayed in Figure 10b are taken from.

**Figure 10:**
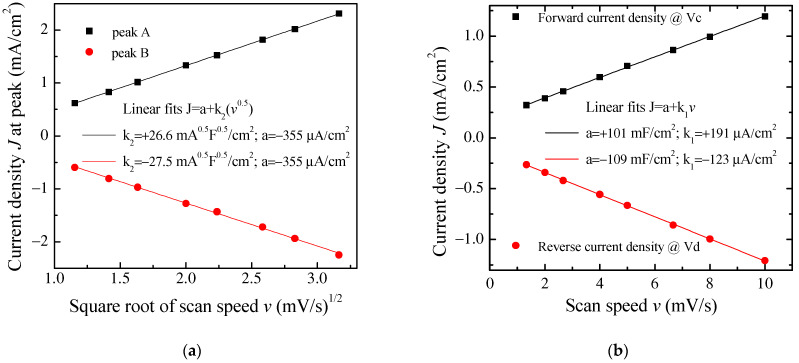
(**a**) Forward (reverse) current density *J* measured at peak A B of Figure 9, plotted as a function of the square root of the scan speed *v*, and linear best-fit data; (**b**) Forward (reverse) current density *J* measured at the voltage V_c_ (V_d_) of Figure 9, plotted as a function of the scan speed *v* and linear best-fit data.

It is worth noticing that the cycles of Figure 9 have the same well-defined current density peaks that are commonly observed when electrodes are tested in an electrolytic solution rather than in a solid-state configuration, as in our case. The presence of the current density peaks when in a solid-state configuration could be related to the role played by DBSA: in fact, the efficiency of the ion diffusion onto the PPY surface may be facilitated in the presence of the finer polymer granules achieved by dispersing PPY into the surfactant and the surfactant, though not covalently bound to the surface of the PPy granules, may electrostatically affect the space charge distribution at the electrodes. It can be noticed that the position of the current density peaks change slightly with the scan speed and that the area enclosed in the current density loops increases as the scan speed increases. Figure 10a shows that the absolute maxima and minima current density, recorded in correspondence to the couple of peaks marked in Figure 9 as A and B, are a linear function of the square root of the scan speed. This kind of relationship is a characteristic feature of the diffusion-limited irreversible redox processes occurring at the electrodes. On the other hand, in the voltage region where the loop current density is relatively flat (see the black vertical lines marked c and d in Figure 9), the current density is found to be linearly related to the scan speed, as shown in Figure 10b. As summarized in the inset of Figure 10b, the absolute value of the slope determined by the best fit curve, which is an estimation of the areal capacitance of the device under test, is of the order of one hundred mF/cm^2^. Based on the results of Figure 9 and Figure 10a,b, the Au/PPy:DBSA/Nafion:LiPs/PPy:DBSA/Au cell has a resistive-capacitive behavior, with capacitance that arises both from the electric double layers that form at the electrodes and from redox transitions at the electrodes. Regarding the former mechanism, the formation of the double later is the expected consequence of interfacing the conducting PPy:DBSA electrode to an electrolyte, that is, the LiPs that impregnate the nafion membrane. The electrochemical activity of the PPy:DBSA electrode can be easily ascribed to polypyrrole, which is a conjugated polymer that exists either in an undoped low electrically conducting state as the reduced form PPY^0^ or in an electrically conducting oxidized state PPy^+^ with the possibility of reversibly changing between the two states under an externally applied voltage. The electrically conducting polypyrrole powder starting from which the PPy:DBSA electrodes are prepared, denoted as X the proprietary organic sulfonic acid used as dopants, can be schematized as (PPy)^+^X^−^. Accordingly, as is usually the case whenever polypyrrole-based electrodes are involved, the redox processes responsible for the current density peaks of Figure 9 may be the result of PPY transitioning between its oxidized and reduced form, which is promoted by the diffusion of lithium ions in and out the polymer surface, as described by the following equations:

(PPy)^+^X^−^ + Li^+^ + e^−^ ↔ (PPy)^0^(LiX)

(PPy)^+^X^−^ + Li^+^ + e^−^ ↔ (PPy)^0^ + Li^+^ + X^−^. (1)

**Figure 11:**
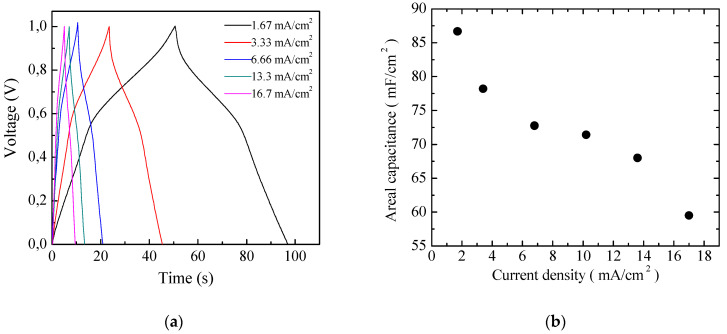
(**a**) Voltage versus time measured in charging–discharging cycles at a constant density on the same Au/PPy:DBSA/Nafion:LiPs/PPy:DBSA/Au cell that Figure 9 and Figure 10 refers to; (**b**) areal capacitance estimated from the charge–dicharge curves of (**a**) as a function of the current density.

The pseudo-capacitive behavior of the Au/PPy:DBSA/Nafion:LiPs/PPy:DBSA/Au cells is evidenced also by the results of the charge–discharge measurements performed in response to the square current density pulses, as shown in Figure 11a. It can be noticed that the voltage profiles measured at different current densities are quite symmetrical and do not follow the linear charge–discharge trend expected in EDLCs [36]. The areal capacitance of the cell, which can be evaluated by means of the charge–discharge curves, is plotted as a function of the current density in Figure 11b. The estimated areal capacitance is approximately 86 mF/cm^2^ at about 1.67 mA/cm^2^, which is in good agreement with the value of about 105 mF/cm^2^ determined by means of the analysis of the current density–voltage loops. The obtained values are of the same order of magnitude as the areal capacitance obtained elsewhere using polyrrole-based electrodes [37,38].

**Figure 12:**
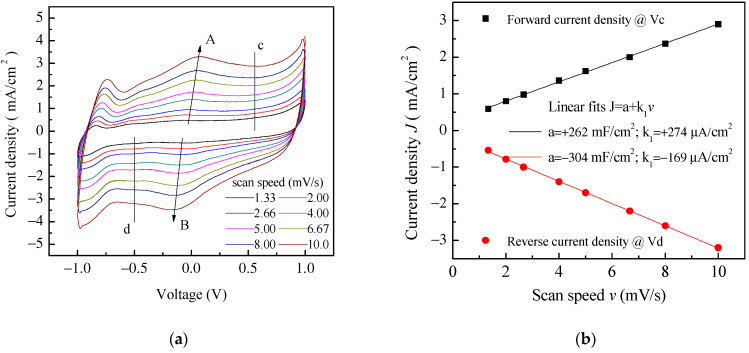
(**a**) Current density–voltage loop of the Au/PPy-GNP:DBSA/Nafion:LiPs/PPy-GNP:DBSA/Au cells measured at different scan speeds. The arrows mark the forward (A) and reverse (B) current density peaks whose position slightly changes with the scan speed. The vertical lines marked c and d in the flat region of the inner loop identify the voltage V_c_ (V_d_) at which the forward (reverse) current density data displayed in (**b**) are taken from; (**b**) Forward (reverse) current density *J* measured at the voltage V_c_ (V_d_) of Figure 11b plotted as a function of the scan speed *v* and linear best-fit data.

**Figure 13:**
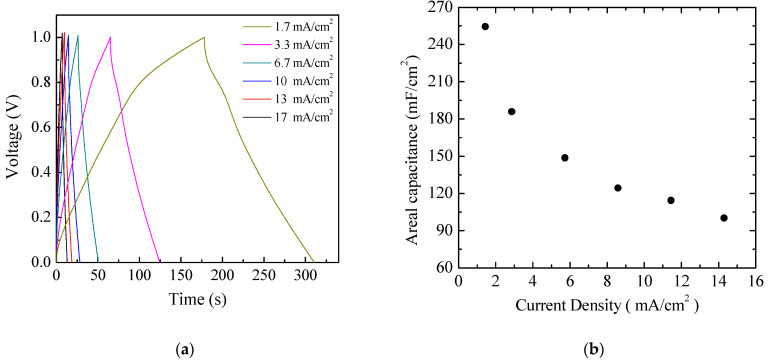
(**a**) Voltage versus time measured in charging–discharging cycles at constant density on the same Au/PPy-GNP:DBSA/Nafion:LiPs/PPy-GNP:DBSA/Au cells that Figure 11 and Figure 12 refer to; (**b**) areal capacitance estimated from the charge–discharge curves from (**a**) as a function of the current density.

Aiming to achieve higher capacitance, planar prototype cells were developed using the three-component ink containing PPy and GNP at a ratio 1:5 by weight. The results of current density–voltage measurements performed on a typical Au/PPy-GNP:DBSA/Nafion:LiPs/PPy-GNP:DBSA/Au cell at different scan speeds are shown in Figure 12a. As expected, the presence of graphene has the effect of increasing the electrode conductivity, as evidenced by the sensitive increase of the current density. In addition, compared to those in Figure 9, the current–voltage loops become closer to the rectangular shape associated with EDLCs with carbon nanostructured electrodes. Nevertheless, a forward and a reverse current density peak identified by the arrows and marked as A and B are broader than those in Figure 9 but are positioned at approximately the same voltage and appear in Figure 12a, meaning that a redox transition involving PPY also occurs in the presence of GNP. While the current density *J* of peaks A and B in Figure 9 is linearly related to the square root of the scan speed *v* in the case of the Au/PPy:DBSA/Nafion:LiPs/PPy:DBSA/Au cell, as expected when diffusion limited redox transitions at the electrodes are involved, any attempt to fit the current density of Figure 12a at zero voltage and at the voltage where peaks A and B from Figure 12 are centered failed unless a linear combination of the kind *J* = a + *k*_1_*v* + k_2_*v*^0.5^ was used. This fact is a confirms that both redox and electric double layer mechanisms are involved in the case of the Au/PPy-GNP:DBSA/Nafion:LiPs/PPy-GNP:DBSA/Au cell at a voltage close to 0. In the voltage regions far from the redox peaks A and B in Figure 12a, the relationship between *J* and the scan speed *v* is expected to become linear. Indeed, the current density evaluated along the lines marked c and d in Figure 12a has a linear dependence on the scan speed, as demonstrated in Figure 12b. The absolute values of the linear best fit of the slope *k*_1_, which are an estimation of the areal capacitance, are 262 mF/cm^2^ and 304 mF/cm^2^, respectively. As a comparison, the areal capacitance was also derived by analyzing the charge–discharge voltage profile, which was measured at different current densities and is shown in Figure 13a. The way that the voltage measured at the Au/PPy-GNP:DBSA/Nafion:LiPs/PPy-GNP:DBSA/Au cell changes with time under a constant current density is closer to the changes observed in carbon-based EDLCs, which are described in Figure 11a, confirming that it is only PPy that is responsible for the faradaic mechanisms from which the capacitance originates. The areal capacitance estimated from the charge–discharge curves from Figure 11a is plotted as a function of the current density in Figure 13b. According to the experimental results, the areal capacitance of the device at about 1.4 mA/cm^2^ is about 255 mF/cm^2^. The values of the areal capacitance of the cells using electrodes developed from the PPy-GNP inks compare well with those obtained elsewhere using polyrrole and graphene based electrodes [39,40].

**Figure 14:**
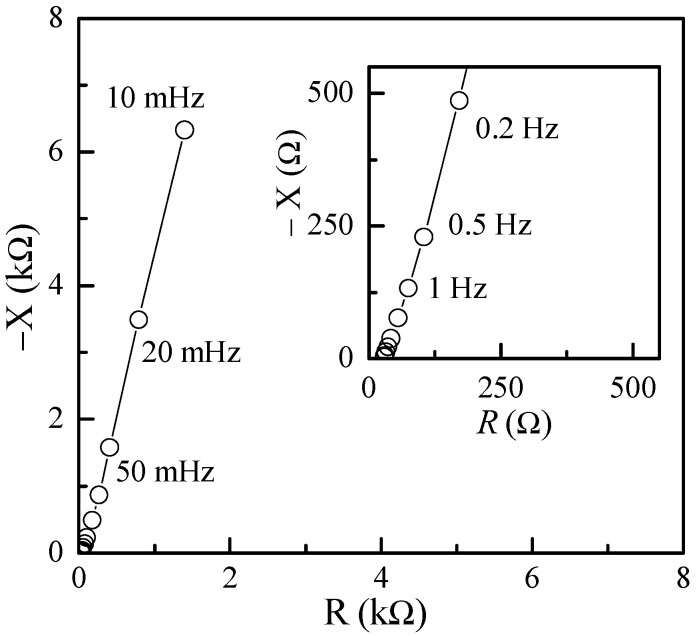
Imaginary vs. real component of the impedance on a typical Au/PPy-GNP:DBSA/Nafion:LiPs/PPy-GNP:DBSA/Au cell at frequencies between 10 mHz and 100 Hz.

Impedance measurements were also performed on the test capacitors using the dedicated instrumentation discussed in [41]. In particular, we explored a very low frequency range, which was as low as 10 mHz with a 0 DC bias and a 20 mV amplitude for the AC test signals. The typical results for these kinds of measurements are reported in Figure 14, where it is apparent that at low frequencies, the equivalent impedance is capacitive in nature. 

**Figure 15:**
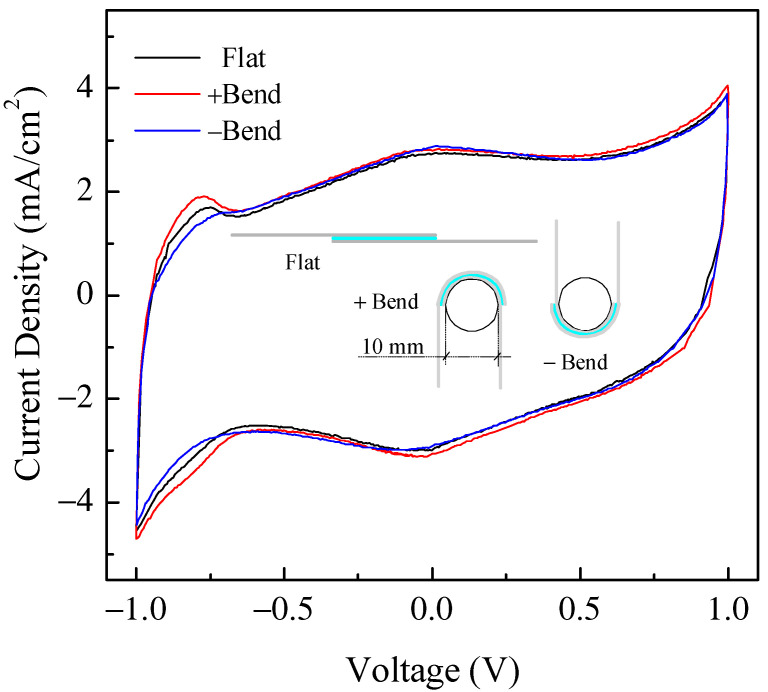
Comparison between current–voltage cycles of the same typical Au/PPy-GNP:DBSA/Nafion:LiPs/PPy-GNP:DBSA/Au cell measured in flat conditions and mounted into the sample holder in Figure 16a with two opposite curvatures.

**Figure 16:**
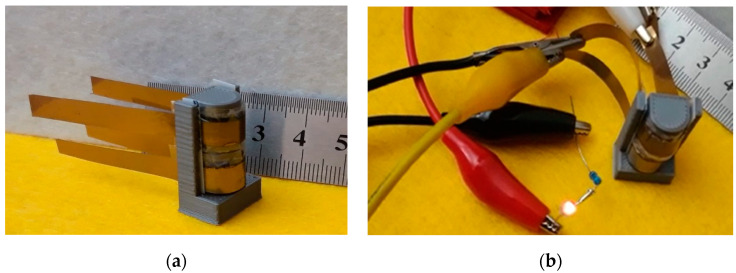
(**a**) Photograph of the sample holder used for bending tests. Two devices, each with an active area of about 1 cm^2^, were inserted into the stage, curvature radius of which was 5 mm; (**b**) picture taken 5 seconds after disconnecting the voltage supply used to charge the two capacitors (in series) inserted into the bending stage to 4 V: the LED remained lit by the charge stored in the devices for several seconds.

Another quite important aspect that needs to be investigated when dealing with flexible devices is, of course, the ability of such devices to retain their characteristics when subjected to bending. In order to demonstrate that the devices we developed here can be regarded as possible candidates for flexible applications, we subjected several samples to the following test procedure: after measuring the current–voltage loop for an as prepared, flat device, we bent the device with the help of a cylindrical stage with a 5 mm curvature radius, and we performed the same current voltage measurements with the device held in this bent position; following the measurements, the sample was removed from the support and was bent the other way around (opposite curvature), and the measurements were repeated once again. Typical results from this measurement procedure are reported in Figure 15. As schematically indicated in the figure, the term “Flat” refers to measurements performed on the “as prepared” device with no bending; the term “+Bend” refers to the bent device with one electrode facing the cylinder; the term “-Bend” refers to the measurements performed when the bending was reversed (the other electrode facing the cylinder). In order to ensure repeatable measurements, the dedicated sample holder reported in Figure 16a was designed and realized. Note that in Figure 16a, the sample holder is shown with two devices inserted (and bent) at the same time. As observed in Figure 15, although changes are certainly noticeable among the three loops, the device essentially retains its ability to accumulate and store charge with similar capacitance in all cases. Clearly, more systematic investigation is required in order to assess the reliability and durability of the devices we have developed these specific bending stress conditions. However, the fact that the devices appear to retain their characteristics when subjected to severe bending stress (with a curvature radius of 5 mm) in both directions can be regarded as quite an encouraging result. 

As a demonstration of the performance of the developed capacitors, the two devices shown inserted into the sample holder in Figure 16a were connected in series and were charged at 4 V. After charging, the voltage supply was disconnected, and the series of a miniature LED with a 1 kΩ resistor was connected in its place, as in Figure 16b, with the LED remaining lit for more than 10 s after the removal of the supply voltage. 

## 5. Conclusions

In this paper, thin films prepared by applying inks developed from commercially available polypyrrole and graphene nanoplatelets were prepared and characterized by means of Raman and FTIR analysis and AFM measurements. Planar supercapacitor prototypes with an active area below ten mm^2^ were also prepared by applying the inks on transparency sheets using an ion-permeable nafion layer impregnated with lithium hexafluorophospate as separator and characterized by means of electrical measurements. The obtained experimental results show that the devices present both pseudocapacitive and electric double layer behavior, with areal capacitance of up to ~250 mF⋅cm^−2^. The commercial availability of the components of these inks, which can be easily applied on to flexible substrates and large areas, together with the possibility of further improvement in terms of the capacitance obtainable by adjusting the concentrations and by optimizing the technology, are promising in view of the application of the developed inks in solid-state flexible supercapacitors. Preliminary experiments show that when bent with a relatively small curvature radius, the structures retain their electrical characteristics. However, further work is required in order to investigate and assess the durability and reliability of these devices under different repeated mechanical stress regimes. 

## Data Availability

Data are contained within the article. The data presented in this study can be requested from the authors.
